# mSphere of Influence: the Young Investigators in Parasitology Meeting—Investing in the Future of Our Scientific Community by Lifting Up and Connecting Junior Faculty

**DOI:** 10.1128/msphere.00615-22

**Published:** 2023-03-09

**Authors:** Monica R. Mugnier, Chi-Min Ho

**Affiliations:** a Johns Hopkins Bloomberg School of Public Health, Baltimore, Maryland, USA; b Columbia University Irving Medical Center, New York, New York, USA; University at Buffalo

**Keywords:** junior faculty, meetings, new PI, starting a new lab

## Abstract

Drs. Monica Mugnier and Chi-Min Ho work in the field of parasitology. In this mSphere of Influence article, they share their experience as cochairs of the Young Investigators in Parasitology (YIPs) meeting, a 2-day biennial meeting for new PIs in parasitology. Setting up a new lab can be a daunting task. YIPS is designed to make the transition a little easier. YIPs is both a crash course in the skills needed to run a successful research lab and a way to build community among new group leaders in parasitology. In this perspective, they describe YIPs and the benefit it has had on the molecular parasitology community. They also provide some tips for building and running a meeting like YIPs, in the hopes that other fields might replicate their model.

## PERSPECTIVE

In 2015, Drs. Bjorn Kafsack, Alexis Kaushansky, Vasant Muralidharan, and Kirsten Hanson, all assistant professors at the time, were having lunch at the annual Molecular Parasitology Meeting (MPM) in Woods Hole when the struggles associated with starting a research lab came up. As Bjorn explains, “I was frustrated that there was no mechanism for transmitting the valuable skills and experiences needed when starting a lab.” This lunchtime conversation was the beginning of the Young Investigators in Parasitology (YIPs) meeting, a 2-day biennial meeting held in conjunction with MPM for PIs in parasitology who are within the first 5 years of starting up their lab. As Alexis Kaushansky noted when we spoke to her and the other YIPS cofounders about starting YIPs, the goal of the meeting was not just to have a discussion of personnel and purchasing issues. It was also to build a culture of collegiality and collaboration among the next generation of PIs.

What Bjorn, Alexis, Vasant, and Kirsten started in 2016 filled a clear need within the community. Since then, YIPS has quickly become an indispensable part of the molecular parasitology community. As the current cochairs for the past two YIPS, we hope to share our experiences organizing this unique meeting in the hopes that the YIPs model can be replicated in other fields to help junior group leaders as they set up their labs.

## WHY YIPS?

Assistant professors are hired for their scientific achievements; most have not received any formal training in logistics, budgeting, procurement, time management, project management, personnel management, information technology (IT), human resources (HR), public relations (PR), mentoring, teaching, negotiation, or difficult conversations (to name a few). All of these skills are crucial for success in the job from day one, and yet, junior PIs are left to master them on the fly, often leading to avoidable early missteps that can have lasting consequences. YIPs was designed to be a quick way to get new PIs up to speed. The meeting brings together a small cohort of YIPs and “veteran PIs” to discuss the transition to independence and share their experiences establishing and running a lab. As cochairs, our goal is to put together a program that provides junior faculty with a crash course in all the skills that are crucial for getting a successful research program off the ground while jump-starting the process of building connections within the community in their new capacity as independent investigators.

## WHO SHOULD ATTEND?

**FIG 1:**
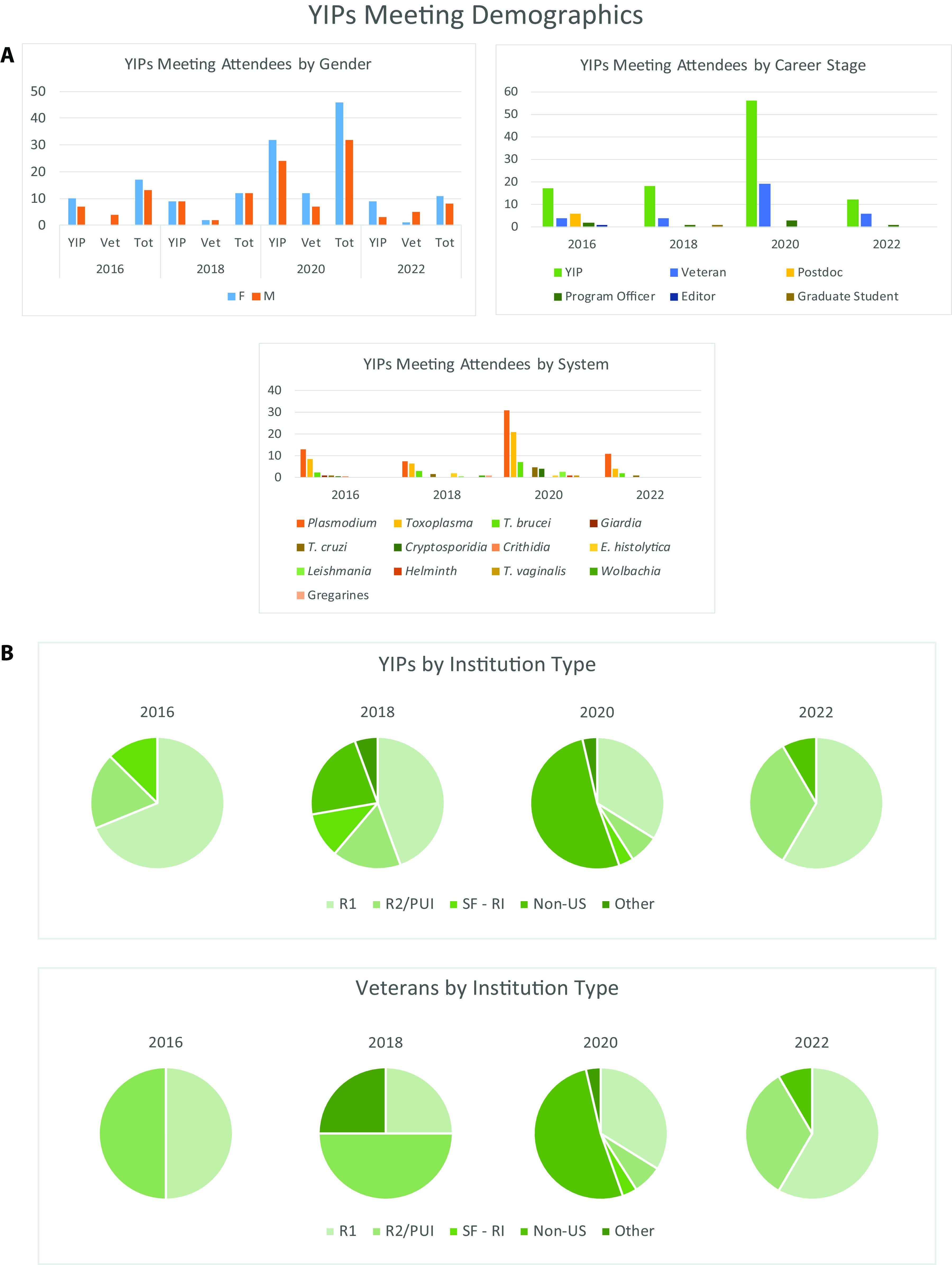
YIPS meeting demographics. (A) Bar graphs detailing YIPS attendee demographics by gender, career stage, and research system. (B) Pie charts detailing YIPS and veteran attendee demographics by institution type. PUI, predominantly undergraduate institution; SF-RI, special focus research institution.

As the YIPs program focuses on the challenges associated with getting a lab up and off the ground, we find that it is most useful for new PIs in the first 5 years of their independent position. A diversity of perspectives, among the YIPs as well as the panel of veteran PIs, is central to creating a vibrant and productive conversation (Fig. 1). Gender and racial diversity within the group are critical to ensure the conversation properly addresses the unique struggles of researchers from underrepresented groups. Similarly, it is vital to include panelists and YIPs from different types of institutions since the unique background and research environment of each investigator influences the way they build and manage a lab. A Howard Hughes investigator running a lab full of postdocs on one of the coasts will almost certainly have a different approach than a PI at a midwestern institution without Ph.D. students. A recent YIPs “alum” will distinctly remember the struggles of setting up a lab, while very senior faculty have the benefit of hindsight and are great at putting things in perspective.

## WHAT TO DISCUSS?

While the details of each new PI’s position are unique, we try to cover the aspects of starting a new lab that are common to all new group leaders. Our current schedule includes discussions of priorities and boundary setting, recruiting, mentoring, project management, publishing, and funding, in addition to a longer session that uses case studies as a basis for discussing the navigation of difficult conversations. We adjust the topics and time spent on each topic depending on the makeup of the group. We’ve found that polling the attendees ahead of the meeting can help to identify topics that are of broad interest and merit a full discussion versus those that are better suited to a small-group lunchtime discussion.

## DISCUSSION FORMAT

Over time, we have experimented with the format of YIPs and found some topics work well as structured panel discussions, while others work better as small-group discussions, case studies, or an “Ask Me Anything (AMA)” question-and-answer session. We’ve tried virtual and hybrid meetings, which have a larger reach, but we feel that the sensitive nature of the conversations at YIPs is best suited to an in-person format. Meeting in person also allows for informal interactions that lead to lasting scientific relationships (lots of coffee breaks are important!).

Structured panel discussions work well for topics that involve a lot of intentional strategies. Veteran PIs often have long lists of tips for new PIs when it comes to things like hiring or publishing—things like the question they ask during every interview or their formula for the perfect cover letter. To keep discussions on track, we like to structure each session around 3 to 5 talking points, depending on the length of the session. These guide the conversation and help keep the focus on the topic at hand while allowing room for the conversation to evolve in a direction that suits the audience. We use a premeeting survey to help identify the group’s most burning questions.

At each YIPs meeting, we recruit program officers from the relevant major funding agencies to provide their perspectives on funding. For these conversations, where attendees often have a lot of specific questions with relatively straightforward answers, an AMA format works really well. This is an efficient way to get a lot of information to the group quickly. Even YIPs without their own questions will learn a lot from listening to the group’s discussion. This session is also a great way to give the YIPs some face time with the program officers in our field.

Finally, case studies work well to help foster conversations around common interpersonal and professional issues encountered while running an academic lab that can be difficult to discuss in the abstract. For example, the question “How do you motivate students?” is so open-ended that the answers tend to be equivalently vague. On the other hand, a detailed case study describing a situation with a student struggling with writer’s block gives the group some details to hang onto, stimulating conversations around the complexity and nuances of mentor-trainee interactions. We’ve found this format works well for the many difficult conversations that new PIs suddenly have to face. As new PIs, many of us spend an inordinate amount of time worrying beforehand about the right way to approach a difficult conversation or afterward about whether we handled it correctly. However, as most veteran PIs are quick to say, there is no single right way to approach a difficult conversation. These case study discussions reveal just how many ways there are to approach a problem and make it clear that the best approach may be different for different people. They also highlight the diversity of ways to frame a problem (e.g., is your issue with a competitor in your department just bad luck, or is it a failure of institutional support?). Finally, these discussions can also alert new faculty to problems they might encounter that aren’t even on their radar yet.

## CONTINUING THE CONVERSATION

Perhaps the most important aspect of this meeting is building community. Our hope is that YIPs will make connections that last well beyond the meeting. To provide a way to continue the conversation, we started ParasiteSlack. In addition to serving as a forum for members of the parasitology community to ask questions and exchange ideas, protocols, and technical and career advice and to advertise job opportunities, ParasiteSlack is a platform for YIPs to keep in touch. We created a private channel for YIPs to keep up an ongoing conversation about all things YIPs. Individual cohorts of YIPs have established their own ways of keeping in touch too.

## KEY TAKEAWAYS

YIPs has been great for our community, making us even more interconnected than we already were and setting a collegial and collaborative tone among new faculty in parasitology. The program is not difficult to build since the relevant topics are already constantly on our minds as new PIs, and the benefits of organizing far outweigh the relatively small amount of work involved. What we’ve learned from organizing YIPs extends well beyond the nuts and bolts of starting a research lab. It has compelled us to reflect on the kind of scientists we want to be and the kind of labs and scientific community we hope to build. If you’d like to try this in your community, here are some things that we’ve learned along the way that may be useful.

Helpful tips!

Before:

- Limiting the meeting to 10–20 YIPS helps foster highly interactive, inclusive discussions.
- Social media and conferences are great venues for advertising the meeting broadly.
- NIH Reporter can be useful for identifying new PIs who might be new to the field.
- Google Polls, conducted among YIPS registrants in advance, are useful for:
  - Identifying your 3–5 talking points for each session
  - Generating a list of questions for the structured panel discussions and AMAs, which you can share with your panelists in advance.

During:

- Think about your goals for each session and actively guide conversations if needed to make sure the key topics of discussion are all covered by the end of the session.
- Interspersing longer coffee breaks and meals liberally between the sessions ensures there are many opportunities for attendees to socialize and build connections.

After:

- Consider making a shared Google Doc to crowdsource tips and takeaways from the discussions.
- After the meeting, share a contact list and set up mechanisms for the group to keep in touch.

